# Challenges and Advantages of Using Spatially Resolved Lipidomics to Assess the Pathological State of Human Lung Tissue

**DOI:** 10.3390/cancers17132160

**Published:** 2025-06-26

**Authors:** Ibai Calvo, Albert Maimó-Barceló, Jone Garate, Joan Bestard-Escalas, Sergio Scrimini, Jaume Sauleda, Borja G. Cosío, José Andrés Fernández, Gwendolyn Barceló-Coblijn

**Affiliations:** 1 Department of Physical Chemistry, Faculty of Science and Technology, University of the Basque Country (UPV/EHU), 48940 Leioa, Spain; ibai.calvo@ehu.es (I.C.);; 2 Health Research Institute of the Balearic Islands (IdISBa), 07120 Palma, Spain; albert.maimo@idisba.es (A.M.-B.); juan.bestard@idisba.es (J.B.-E.); jaume.sauleda@ssib.es (J.S.); borja.cosio@ssib.es (B.G.C.); 3 Research Unit, Hospital Universitari Son Espases, 07120 Palma, Spain; 4 Department of Respiratory Medicine, Hospital Universitari Son Espases, 07120 Palma, Spain; 5 Centro de Investigación Biomédica en Red in Respiratory Diseases (CIBERES), 28029 Madrid, Spain

**Keywords:** lipidomics, arachidonic acid, glycerophospholipids, lipids, phospholipids, lung cancer, matrix-assisted laser desorption-ionization mass spectrometry, mass spectrometry imaging

## Abstract

**Background:** Mass spectrometry imaging (MSI) lipidomics is a subset of spatially resolved techniques wherein lipids are detected by mass spectrometry, allowing their multiplexed detection and acquiring position-correlated spectra along a tissue section. Rapid advances in the field provide solid evidence demonstrating how specific and regulated lipid distribution is in any biological context. **Objectives**: Herein, we describe the MSI, particularly matrix-assisted laser desorption/ionization (MALDI-MSI), challenges and advantages in defining human lung pathophysiology, particularly in lung cancer and chronic obstructive pulmonary disease, leading causes of death. **Methods**: MALDI-MSI analysis of lung tissue sections at 25 μm of lateral resolution allowed associating specific lipid profiles with the main tissues present and independently assessing the impact on lipid composition of smoking, chronic inflammation, and lung cancer. **Results**: Consistent with MALDI-MSI studies in tumor epithelia, arachidonic acid-containing phospholipids increased, agreeing with its role as a precursor of numerous bioactive molecules participating in cell differentiation and malignization. Next, a gene expression dataset of epithelial human non-small cell lung cancer samples was analyzed using system biology approaches, revealing that, consistent with the most relevant changes in lipid profiles, the network dominated by the tumor-associated module included genes tightly involved in phosphatidylinositol and sphingolipid metabolism. Hence, despite the intrinsic difficulties entailed by lung tissue handling, the results strongly encourage future analysis at higher lateral resolutions so that the lipidome changes associated with each lung cellular type, even subtype, could be fully mapped. Therefore, MALDI-MSI lipidomics definitively broadens the options, some still rather unexplored, to delve into pathophysiology at the cell-type level.

## Simple Summary

Mass spectrometry imaging (MSI) lipidomics is a cutting-edge technique that maps the spatial distribution of hundreds of lipids within a tissue section. We used MSI to study the lipid patterns found in the human lung, in particular in two important pathological conditions: lung cancer and chronic obstructive pulmonary disease. We could associate differential lipid profiles with the main tissue types found in the lung thanks to the spatial resolution used: 25 μm. The main result in tumor tissue was the accumulation of lipids containing arachidonic acid, a precursor to molecules that actively participate in cell differentiation and cancer development. We also used public databases of lung cancer studies and established that lung cancer impacts two different families of lipids: phosphatidylinositol and sphingolipids. Overall, this study underscores the great potential of MSI lipidomics to provide underexplored insights into lung diseases.

## 1. Introduction

**Figure 1:**
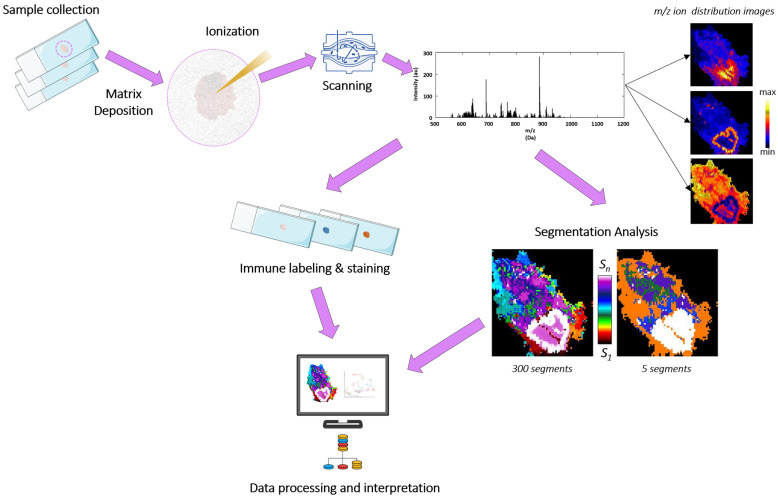
Typical MALDI-MSI workflow in a biomedical context. A standard MALDI mass spectrometry imaging (MALDI-MSI) protocol in biomedical applications involves three key phases: Sample Preparation: Thin tissue sections from human biopsies are cryopreserved and coated with a crystalline organic matrix; Spectral Acquisition: The prepared samples are systematically scanned using a high-resolution mass spectrometer to generate spatial molecular profiles; and Multimodal Integration: Computational alignment of MSI segmentation maps with histochemical stains (e.g., H&E) and targeted protein localization data from immunohistochemistry/immunofluorescence enables precise demarcation of both morphological landmarks and disease-specific zones (adapted from Maimó-Barceló et al. [7]).

Single-cell technologies are finally making it possible to capture the cellular complexity of any tissue. However, transcriptomic and metabolomic programs are highly dependent on both cell-to-cell and cell-to-environment interactions. Thus, it seems that spatially resolved molecular profiles will be critical for identifying molecular drivers of cellular interactions in both physiological and pathological conditions [1]. In this context, mass spectrometry imaging (MSI) techniques are a subset of spatially resolved techniques wherein the analytes are detected based on mass spectrometry, which is the best in terms of high sensitivity and specificity, high throughput, and high accuracy [2]. MSI enables the multiplexed detection and localization of biological molecules by acquiring position-correlated spectra along a tissue section [3,4]. Once spectra are acquired, specialized software transforms the variable “peak intensity” of a particular compound into a pixel colored according to a color scale, one per coordinate, generating a final image of its distribution (Figure 1). MSI methodology can be applied to analyze proteins, metabolites, or lipids (Supplementary Figure S1), providing high-quality molecular data and connecting both pathology and mass spectrometric analysis to tissue-based research. There are several MSI techniques, but MALDI (matrix-assisted laser desorption/ionization), which uses soft ionization to extract the analytes from the tissue, is currently the most widely used to analyze lipid profiles [5,6]. One of the key parameters in MSI analysis is the lateral (or spatial) resolution achieved, which is determined by laser spot size and the distance the laser moves between one analysis zone and the next. Most current MALDI-MSI equipment allows reaching 50 to 10 µm of lateral resolution; consequently, the molecular information gathered from the tissue is close to single-cell.

MALDI-MSI has provided a new level of understanding by clearly showing the specific spatial distribution of lipid species in tissues (Figure 1) and also that lipid profiles are tightly linked to cell-type identities and, even more importantly, their precise pathophysiological states [8,9,10]. While this precision prompted the lipid analysis of a wide variety of tissues by spatially resolved techniques, few studies used human lungs [11,12]. This is notable because the lungs are affected by two of the deadliest diseases: lung cancer and chronic obstructive pulmonary disease (COPD) [13,14]. Thus, lung cancer remains the leading cause of cancer-related deaths, accounting for over 1.8 million deaths and 2.1 million new cases in 2020 alone [14], while COPD is the third leading cause of death worldwide [13]. In addition, COPD increases the risk of lung cancer, and around 40 to 70% of lung cancer patients are diagnosed with COPD [15,16]. Herein, we focused on these two diseases, analyzing lung tissue sections at a lateral resolution of 25 μm and comparing them to the non-affected counterpart and healthy tissue. At this resolution, we were able to associate specific lipid profiles with the main tissue types and independently assess the impact of smoking, chronic inflammation, and tissue malignization. Consistent with numerous studies analyzing tumor epithelia, the most robust change was the increase in arachidonic acid (AA)-containing phospholipids, in agreement with the role of this fatty acid in cell differentiation as well as cell malignization [8,9]. Despite the intrinsic difficulties that the handling of lung tissues entails, the results strongly encourage the analysis of the tissue at higher lateral resolutions to fully map the changes in lipid composition associated specifically with each lung cellular type and subtype. There is no doubt that the rapid and constant advances in MSI techniques offer a unique opportunity to reach this ambitious aim.

## 2. Materials and Methods

### 2.1. Study Design

Consecutive smoker and non-smoker patients undergoing bronchoscopy for diagnosis purposes were invited to participate. Written informed consent was obtained for each patient recruited at Hospital Universitari Son Espases (Palma, Balearic Islands, Spain). Patients underwent conscious sedation during fiber-optic bronchoscopy, which was performed for diagnosis purposes in either the right or left lung. Bronchial biopsies were performed with alligator forceps in the second or third sub-segmental division of the right or left lower lobes in the COPD and non-smoker control (hereafter, Control) groups. In patients with endobronchial lesions, later confirmed as neoplasms, a biopsy was taken from the tumoral lesion (labeled as tumor) and another biopsy from the opposite lung to the neoplasm (labeled as non-tumor). Biopsies were immediately snap-frozen in liquid nitrogen and saved at −80 °C until analysis.

All patients underwent lung function assessment by means of forced spirometry. Patients were then classified based on the results of bronchoscopy, chest computed tomography, and spirometry as COPD, lung cancer, smoker, or control.

Tissue sections approximately 10 μm thick were prepared in a cryostat (Leica CM3050S, Leica Biosystems, Wetzlar, Germany) at −20 °C without the use of cryoprotective substances or embedding material and mounted on regular glass microscope slides. To facilitate structural identification, the consecutive section was stained with hematoxylin and eosin (HE, SigmaAldrich, Madrid, Spain).

### 2.2. Sample Preparation for MALDI-Imaging, Measurement Conditions, and Data Analysis

Tissue sections were coated with 1,5-diaminonaphthalene (DAN, 97%, Sigma-Aldrich Chemie, Steinheim, Germany), a suitable matrix for negative-ion detection [17]. To help structure identification, a consecutive section was stained with HE. Leucine enkephalin acetate hydrate, ammonium acetate, and sodium hydroxide solution were purchased from Sigma Aldrich Chemie (Steinheim, Germany).

Garate et al. describes in detail MALDI imaging and data analysis [18,19]. Briefly, a total of 23 sections were scanned in negative-ion mode using a MALDI-LTQ-Orbitrap XL (Thermo Fisher Scientific, San Jose, CA, USA), an orbitrap analyzer equipped with an N_2_ laser (LTB, Berlin, model MNL 100), 100 µJ max power, an elliptical spot, and a 60 Hz repetition rate. The mass resolution used to record the data was 60,000 at *m*/*z* = 400 Da. The scanning range was 550–1000 Da. Within this *m*/*z* range, we focused on glycerophospholipids and sphingolipids detected more consistently in the negative mode, namely PE, ether PE, PI, PS, and SM. For each pixel, two microscans of 10 laser shots were recorded, and the raster size used was 25 μm. We used in-house programs developed in MATLAB (MathWorks, Natick, MA, USA) to align and analyze spectra.

The MSI data processing protocol may be found in ref. [19]. In brief, spectra were normalized using a total ion current algorithm and aligned using the parsing stage the Xiong method [20]. To streamline analysis, peaks with intensity values below 0.5% of the intensity of the strongest peak were filtered, reducing the number of *m*/*z*. Lipid assignments were made by matching experimental *m*/*z* values and the species in the software’s database (<33,000 lipid species plus adducts) and in the LIPID MAPS^®^ database (www.lipidmaps.org (last accessed on 17 March 2025)). Mass accuracy was established <9 ppm and typically <3 ppm.

For each experiment, the spectra of each tissue segment was obtained using hierarchical divisive-rank compete analysis (HD-RCA) as an MSI segmentation algorithm [21]. The specific nature of the bronchial tissue type associated with each of the MSI segments was identified and confirmed by at least two expert pathologists working at the hospital where the patients underwent the bronchoscopic procedure.

The percentage of each lipid species was calculated by dividing its intensity by the total intensity of all species within the same lipid class (e.g., for PE species: PE_i_% = I_PEi_/ΣI_PE_·100). This method enables averaging the lipid profiles across sections within the same study group or comparisons between multiple study groups. To be able to delve into the potential biological meaning of the results and the potential fatty acid moieties involved, we compared the phospholipid profiles obtained with those previously published [22,23,24]. Hence, we will describe species as “highly enriched” in a particular fatty acid only when this characterization is supported by published data. Finally, the compounds identified as plasmalogens of PE (PE P) have the same *m*/*z* values as their corresponding alkyl-ether analogs (PE O) with an additional double bond. In the graphs we have assigned them to PE P for simplicity, but they are included in the Supplementary Tables S1–S3. This decision is based on the following rationale. On the one hand, in most of the tissues analyzed in the literature, the content of alkenyl-PE is substantially higher than that of alkyl-PE. On the other hand, the resultant species profile fits with the published literature. Nevertheless, the presence of additional isobaric lipid species sharing the *m*/*z* cannot be ruled out.

### 2.3. Gene Set Enrichment Analysis

The GSE168466 dataset from Gene Expression Omnibus (GEO) was used to explore the difference in gene expression level between healthy and tumor lung epithelial cells. The dataset contains RNA-seq information on human epithelial cells from non-small cell lung cancer tissue (*n* = 49) and its healthy counterpart (*n* = 49). Weighted correlation network analysis (WGCNA) was used to identify correlation patterns of gene expression across samples using the CEMiTool package, which provides a comprehensive set of functions for WGCNA [25]. The parameters were set as follows: (1) the most variant genes were selected according to a *p*-value cutoff of 0.1 and (2) Pearson’s correlation was used to identify the modules of co-expressed genes, using a soft-thresholding power automatically set to ensure scale-free topology, with a β-value of 7 (scale-free R^2^ = 0.81). The minimum module size was set at 20 genes, and similar modules were merged. Pathway enrichment was calculated with EnrichR [26] using the Reactome database (2022). The top 10 pathways were ranked and plotted according to *p*-value enrichment and considered representative of the modules included. Lipid genes listed in co-expressed modules were analyzed with the STRING tool [27]. The default combined string score represents node–strength interaction, and enrichment analysis was calculated to illustrate node contribution to the lipid pathway. Cytoscape v3.10.2 was used to color nodes according to co-expression modules, calculate network enrichment using the String enrichment app, and generate the final network layout.

## 3. Results

### 3.1. MALDI-MSI Lipid Segments Established Accurately Described the Tissue Architecture Found in Bronchoscopic Biopsies

Twenty subjects were recruited, and samples were classified into the following groups: patients with lung cancer (tumor samples (*n* = 5) and non-tumor samples (*n* = 5)), patients with COPD (*n* = 4), smokers (*n* = 5), and non-smokers with normal lung function (*n* = 4, control group). The characteristics of the cohort are summarized in Table S1.

**Figure 2:**
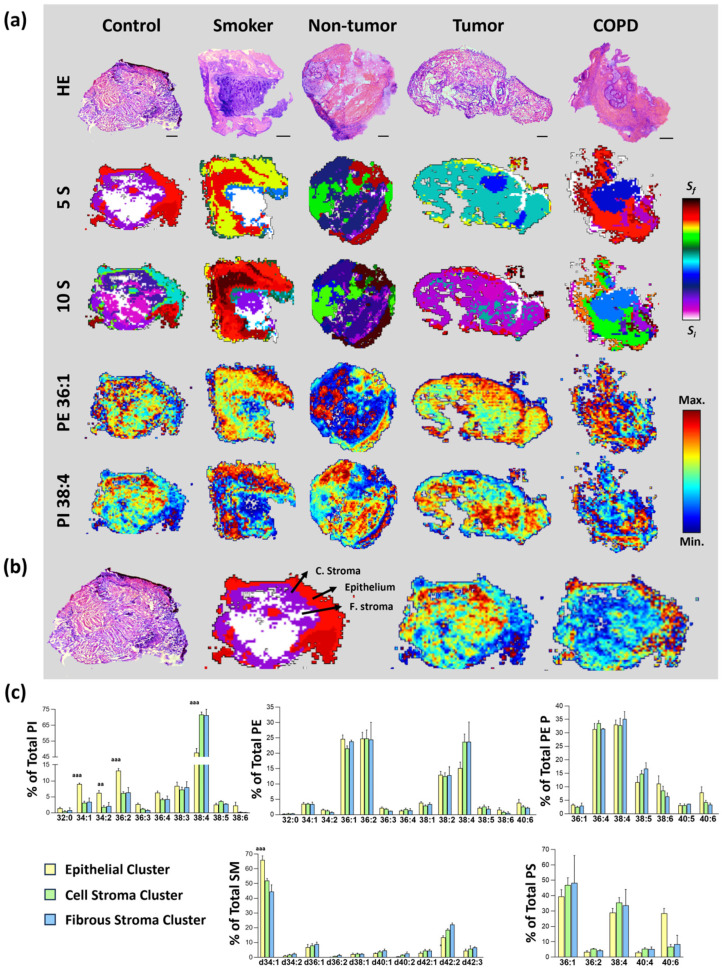
Differences in membrane lipid profile between lung epithelium, cellular, and fibrous stroma. (**a**) First row, HE image of the consecutive section obtained from bronchoscopy biopsies. Scale bar = 150 μm. Second and third rows, segmentation images generated using non-supervised HD-RCA segmentation establishing 5 and 10 segments. Fourth and fifth rows, distribution of selected lipid species. (**b**) HE-stained section with the tissue types identified (E: epithelium, CS: cellular stroma, FS: fibrous stroma), segmentation images associated with E (light red cluster), CS (purple cluster), and FS (white cluster), and images of selected *m*/*z*. (**c**) Bar diagrams showing the comparison of the lipid profiles of the three main clusters. Values are expressed as % of total membrane lipids (mean ± SEM), *n* = 3. Statistical differences were assessed by multiple unpaired *t*-tests; aa, *p* ≤ 0.01; aaa, *p* ≤ 0.001.

Bronchoscopy biopsies usually consist of respiratory mucosa, including epithelium and stroma, and, occasionally, cartilage (Figure 2a). In turn, bronchi have cartilaginous reinforcement and a pseudostratified columnar epithelium, which is highly heterogeneous in composition and function, containing ciliated, goblet, club, and underlying basal cells [28]. The heterogeneity described at the structural and cellular level posed a real challenge during the MALDI-MSI image interpretation.

In Figure 2a, a series of MALDI-MSI images of a representative section of each study group was compiled. We used a segmentation algorithm (HD-RCA) to sort and group the pixels with the most similar lipid profile which, in the end, constitutes a lipid segment or cluster. It is important to bear in mind that segment generation is a mathematical process, and the number of segments can range from one to virtually the number of pixels the image is composed of. So, as a rule of thumb, we established as many segments as biological significance we can ascribe. Due to a lack of previous knowledge on lung epithelial lipidome, we took a prudent approach while establishing the epithelial segment and used just one. Nevertheless, considering that the segment can be further divided, it is highly plausible that analysis at higher lateral resolution would help identify additional segments accounting for the presence of epithelial subtypes, similar to that observed in colon epithelial cells during differentiation [8,9].

In bronchoscopy biopsies of the control group, we were able to identify three segments that matched the epithelium, the cell stroma, and the fibrous stroma (Figure 2b). Interestingly, the lung stroma preserved one of the characteristics of colon stroma: the content of AA-containing lipid species in stromal phosphatidylinositol (PI) and phosphatidylethanolamine (PE) was at least 2-fold higher than in the epithelium (PI 38:4 and PE 38:4, Figure 2c) [8]. Conversely, stromal clusters contained lower levels of species highly likely to be enriched in docosahexaenoic acid (DHA, 22:6n-3) according to the literature, such as PE 38:6, PE 40:6, PE P 38:6, PE P 40:6, and PS 40:6 [22,23,24], which, depending on the species, decreased by 30 to 90%.

### 3.2. Identification of a Tissue-Type Dependent Response to Lung Malignization at the Lipid Profile Level

**Figure 3:**
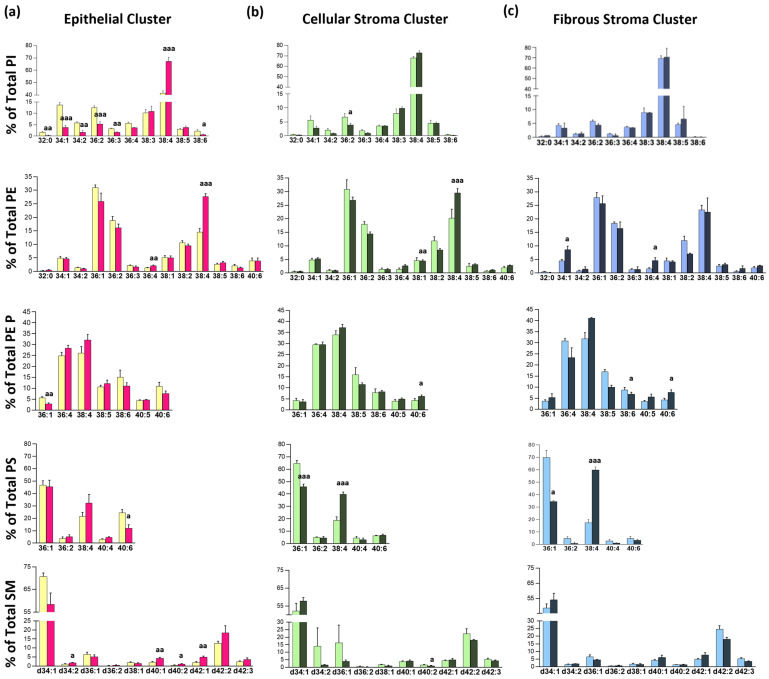
Impact of lung cancer on lung tissue membrane phospholipid profile. Bar diagrams show the comparison of non-tumor and tumor lung tissues: PI, PE, PEP, SM, and PS composition in each of the clusters identified (from left to right): (**a**) epithelium, (**b**) cell stroma, and (**c**) fibrous stroma. Values are expressed as % of total membrane lipids (mean ± SEM) in non-tumor (lighter colors, *n* = 4–5 E; *n* = 3–4 CS; *n* = 3–4 FS) and tumor tissue (darker colors, *n* = 4–5 E; *n* = 5–6 CS; *n* = 2 FS). Statistical differences were assessed by multiple unpaired *t*-tests. a, *p* ≤ 0.05; aa, *p* ≤ 0.01; aaa, *p* ≤ 0.001. Detailed results are included in Table S3.

It is well established that tissue neoplastic transformation induces profound changes in cells at both gene and metabolic levels. Numerous studies have demonstrated the impact of the malignant transformation on the lipidome in a wide range of cancers, including lung cancer [11,29,30]. We used MALDI-MSI to study the impact of cancer at the tissue-specific level, avoiding the bias induced by tissue or cell-type isolation protocols. Thus, we first compared the lipid profiles of the malignant and non-malignant lung tissues obtained from patients with lung cancer (Figure 3).

Globally, the most consistent change in the lung epithelium was a robust increase in all the lipid classes detected in species highly likely to be enriched in AA [22,23,24]. The greater impact was in diacylglycerophospholipids (PI, PE, and PS lipid classes, approx. 60%) than in PE plasmalogens (PE P, approx. 18%). These observations were consistent with the literature and our previous results in the colon epithelium [8,9]. Thus, the lung PE profile was enriched in 36:1, 36:2, 38:2, and 38:4 species, each accounting for 10–20% of total PE, which were the most altered species in the tumor epithelium. While 38:4 and 36:4 content drastically increased by 91.3% and 64.4% in the malignant tissue compared to non-malignant tissue (14.5 vs. 27.7% and 1.3 vs. 2.2%, respectively), PE 36:1 and PE 36:2 content tend to decrease by 16.4% and 14.4%, respectively (31.0 vs. 25.9% and 18.8 vs. 16.1%). PE plasmalogen species showed a similar trend, that is, an increase in AA- and a decrease in MUFA-containing species, but reached significance only for PE P 36:1, which decreased by 49.5%. The decrease observed in DHA-containing species, PE P 38:6 and PE P 40:6, was also particularly interesting, albeit not significant.

Generally, PI 38:4 is considered the most abundant PI species [31]. However, similar to the colon epithelium, the lung epithelium PI repertoire was enriched in more species, such as PI 34:1, PI 34:2, PI 36:2, PI 36:4, and PI 38:3, each of which accounted for 5 to 10% of total PI. The results indicated that lung cancer induced a profound remodeling in PI species in the non-tumor epithelium. Thus, in the tumor epithelium, PI 38:4 content increased by 62.1% compared to the control epithelium (41.5 vs. 67.3%), while PI 36:2, PI 34:2, PI 34:1, and PI 32:0 content decreased by 57.5, 69.2, 72.1, and 81.6%, respectively (12.5 vs. 5.3%, 5.9 vs. 1.8%, 13.7 vs. 3.8%, and 1.8 vs. 0.3%). Consistent with changes in PE and PI, PS 38:4 content tended to increase (49.1%) in the tumor epithelium compared with NT, while PS 40:6 (enriched in DHA) significantly decreased by 50.8%. Finally, SM d34:1, the most abundant SM species, decreased by 17.3% in tumor tissue (70.7 vs. 58.5%).

Next, we explored the differences in the remaining tissues. In most of the samples, the pathologists identified two types of stroma, cellular and fibrous stroma, which differ in the cell content. However, only PS showed a robust increase in PS 38:4 and a drastic decrease in PE 36:1, which was sharper in fibrous than in cellular stroma. PE 38:4 also increased in cellular but not in fibrous stroma, most probably due to the lower content of immune cells in the latter.

Altogether, these results indicated that the lung epithelium was the most affected tissue in terms of lipid composition, showing a solid increase in AA-containing species throughout all the lipid families detected.

### 3.3. Impact of Chronic Inflammation on Healthy Lung Tissue

**Figure 4:**
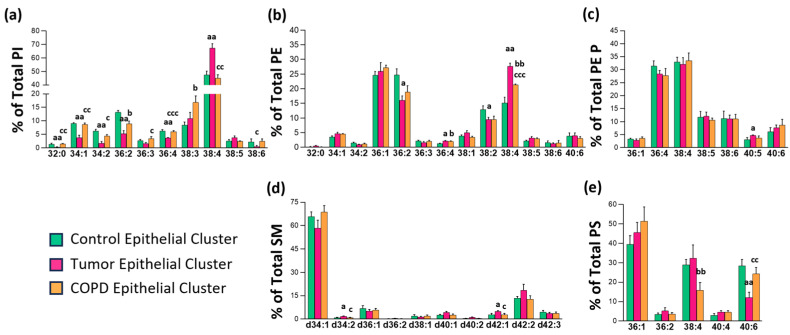
Impact of chronic inflammation (COPD) and lung cancer on the lung epithelium membrane lipid profile. Bar diagrams show the comparison of epithelial (**a**) PI, (**b**) PE, (**c**) PEP, (**d**) SM, and (**e**) PS composition in the control (C), tumor (T), and COPD groups. Values are expressed as % of total membrane lipids (mean ± SEM) in C (*n* = 4), T (*n* = 5), and COPD (*n* = 4) groups. Statistical differences were assessed by multiple unpaired *t*-tests: a, C vs. NT; b, C vs. T; c, NT vs. COPD. Depending on the figure one, two, and three symbols may represent, *p* ≤ 0.05, *p* ≤ 0.01, and *p* ≤ 0.001, respectively. Detailed results are included in Table S3.

Chronic inflammation is associated with a higher risk of developing cancer [15]. For this reason, we were interested in assessing the differences in lipid composition of tissues obtained from patients with COPD and lung cancer and comparing them to the healthy control group (Figure 4). Interestingly, the most affected lipid classes were PI and, to a lesser degree, PE, while in PE P, SM, and PS, changes were scattered. Compared to the control group, COPD showed lower levels of PI 36:2, PE 36:2, PE 38:2, and PS 38:4 (32.4, 23.3, 26.0, and 44.9%, respectively) and higher content of PI 38:3 and PE 38:4 (99.0 and 40.3%, respectively). Consistently, PE 38:4, which is also an increase in tumor tissue (82.3%), was one of the species that clearly increased in the colon epithelium of patients with chronic inflammation [10] and adenomatous polyps [8]. The comparisons of the cellular and fibrous stroma lipid profile can be found in Table S3.

### 3.4. Changes in Lipid Composition in Non-Malignant Tissues

**Figure 5:**
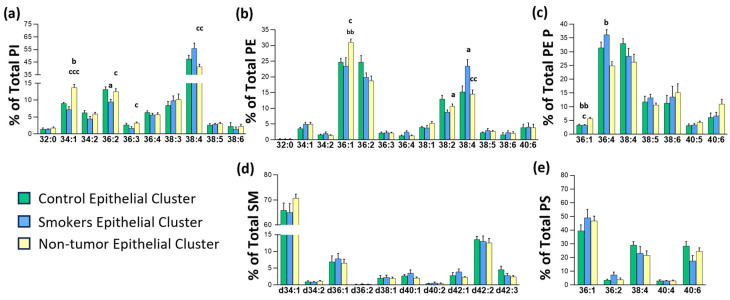
Non-tumor lung epithelial membrane phospholipid profile comparison. Bar diagrams show the comparison of epithelial (**a**) PI, (**b**) PE, (**c**) PEP, (**d**) SM, and (**e**) PS composition in the control (C), smokers (Sm), and non-tumor (NT) groups. Values are expressed as % of total membrane lipids (mean ± SEM) in C (*n* = 4), Sm (*n* = 5), and NT (*n* = 5). Statistical differences were assessed by multiple unpaired *t*-tests: a, C vs. Sm; b, C vs. NT; c, and Sm vs. NT. One, two, and three symbols represent, *p* ≤ 0.05, *p* ≤ 0.01, and *p* ≤ 0.001, respectively. Detailed results are included in Table S3.

Finally, we investigated the changes in the lipid composition in tissues not affected by chronic inflammation or cancer, wherein macroscopic changes, if any, are subtle, by comparing the control, smoker, and non-tumor groups (Figure 5). The most consistent changes occurred in the epithelium, particularly in PI, PE, and PE P. Conversely, the changes followed similar patterns in cellular and fibrous clusters and differed from those in the epithelium (Table S3).

In the epithelium, the PI profile in the control and smoker groups was quite identical, while statistical changes were mainly identified when compared with the non-tumor group. The largest change occurred in PI 38:4, decreasing by 12.8 and 25.7%, while PI 34:1 increased by 51.9 and 90.6% (control vs. non-tumor and smoker vs. non-tumor, respectively). Conversely, PE composition in the control and smoker groups showed some changes, such as increased PE 38:4, despite it reaching significance only for PE 38:2 (Figure 5, Table S3).

### 3.5. Lipid Metabolism Gene Expression Regulation in Non-Small Cell Lung Cancer

To delve into the biological significance associated with the changes observed in malignant epithelia, we used the gene expression dataset GSE168466 containing healthy and tumor epithelial human non-small cell lung cancer samples of 98 patients. These samples were classified by the authors of the data based on the WHO basis for histological morphology and molecular typing, based on labeling by immunohistochemical P63/Napsin A and TTF-1/CK7 double staining, and were divided into the following five groups according to tissue origin: bronchoalveolar carcinoma, alveolar epithelial carcinoma, bronchial epithelial carcinoma, secretory adenocarcinoma, and mucinous adenocarcinoma.

**Figure 6:**
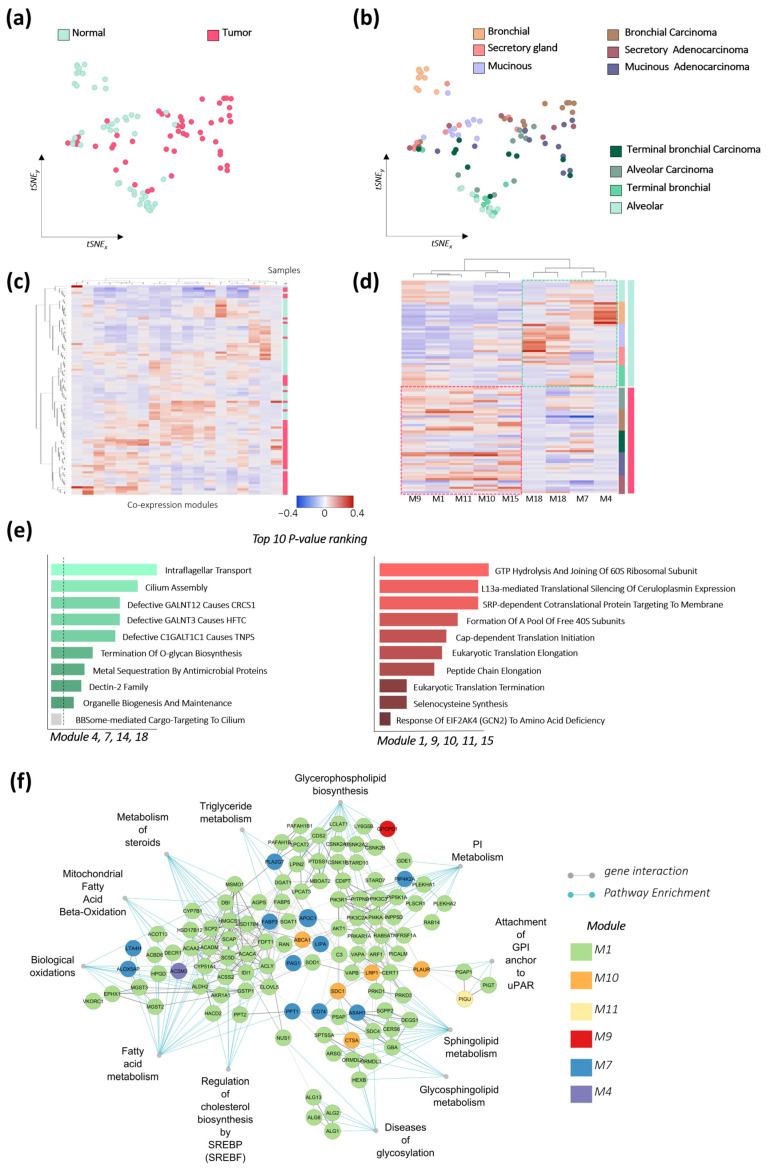
Lipid metabolism network associated with lung epithelial malignization. WGCNA was used to identify co-expressed genes representative of lung epithelium malignization based on the gene dataset GSE168466. (**a**,**b**) tSNE plot summarizing sample distribution according to (**a**) their co-expression module profile and pathological state (healthy group, green dots; Tumor group, red dots), and (**b**) lung region and epithelial tissue of origin; (**c**) hierarchical-clustering heatmap of module eigengene highlighting the association of the module to epithelial malignancy; (**d**) modules more differentially expressed in healthy or tumor samples were selected based on ranked ANOVA score, representing profiles that better described epithelial transformation; (**e**) pathway enrichment analysis computed using EnrichR for healthy (green plot) and tumor modules (red plot) describing the top 10 *p*-value ranked Reactome pathways; (**f**) STRING network representing the overall interaction of genes involved in lipid metabolism.

First, we conducted a Weighted Gene Co-expression Network Analysis (WGCNA) to identify modules of co-expressed genes associated with epithelial malignization [32]. Such a strategy is useful for uncovering unexpected patterns and connections that can be easily overlooked using more targeted approaches. WGCNA application of omic and multiomic data integration to phenotypic information has been applied in fields covering the discovery of regulatory programs, target genes, proteins, and metabolites involved or associated with specific phenotypes [33,34]. The WGCNA was calculated using the CEMiTool package [25], which returned 20 modules using automatized determination of beta value (β = 7, r^2^ = 0.81) to ensure network scale-free topology. In Figure 6a, the tSNE plot summarizes gene expression variance according to the profile of co-expression modules for each sample (module eigengene). Generally, healthy and tumor samples show several expression modules, demonstrating an opposite distribution (green vs. red). The sample distribution also conveyed lung region and tissue of origin (right side of the tSNE plot). Similarly, the hierarchical-clustering heat map of the eigengene modules arranged the samples according to pathological state (Figure 6b).

Then, we ranked the modules according to the difference between healthy and tumor samples and selected those with the best scores. Thus, nine up- and down-regulated modules were identified in the healthy vs. tumor comparison (Figure 6c). Modules 4, 7, and 18 were more correlated to healthy samples, which showed significant enrichment for processes related to the cilium assembly and termination of O-glycan biosynthesis. Conversely, tumor samples showed a positive correlation for modules 1, 9, 10, 11, and 15. The pathway enrichment analysis described protein metabolism processes such as GTP hydrolysis and joining of the 60S ribosomal subunit and SRP-dependent co-translational protein targeting to the membrane, among others (Reactome 2023.1.Hs; Enrichr) (Figure 6d) [26].

Module composition was further examined using the Reactome database, identifying the lipid-related genes included as well as the most representative pathways according to the enrichment analysis (Figure 6e). The network was computed using the subtracted genes as input in the STRING database [27] and the combined string score was used to define the strength of node interaction. Overall, the network is dominated by the tumor-associated module 1. This module includes a set of lipid-related genes tightly involved in phospholipid metabolism (CSNK2A1, CDIPT, PI4KA, GDE1, LPCAT3, LPIN2, PIK3C3, LPCAT2, CSNK2A2, PIK3C2A, RAB5A, MBOAT2, LCLAT1, STARD10, STARD7, PIP5K1A, PLEKHA1, RAB14, CSNK2B, INPP5D, CDS2, PIK3R1, PTDSS1, ARF1, PLEKHA2, DGAT1, and PITPNB) and, more specifically, in PI and in sphingolipid metabolism (ORMDL2, SPTSSA, SGPP2, DEGS1, VAPA, PRKD3, CERS6, ORMDL3, PRKD1, and VAPB). The alteration in these pathways would be consistent with the most relevant changes in lipid profiles.

## 4. Discussion

In this study, we reinforce the current evidence demonstrating the great potential of MALDI-MSI as a powerful tool for detecting and localizing specific lipid profiles within biological tissues, making MALDI-MSI a valuable approach to providing new insights into complex biological systems, such as the pathophysiology of the respiratory system. While these techniques generate comprehensive information about tissue composition, drawing the correct functional conclusions about tissue or organ behavior is delicate due to the high heterogeneity coexisting at the cell compositional level [28,35,36,37]. Further, MSI techniques are challenging due to the vast and specific amount of data generated, demanding a reinterpretation of lipid metabolism and highlighting the gap in knowledge regarding the molecular mechanisms tailoring the precise lipid profile [38].

MSI clearly establishes that the lipid composition is highly cell type-specific but also very sensitive to the cell physiopathology state. Spatially resolved techniques have been used to analyze an extensive list of human tissue lipidomes, including brain [39,40], skin [41,42,43], kidney [44], colon [8,9], or breast tissue [45,46], all revealing the tissue-dependent distribution of lipid profiles. Curiously, information about human lung composition at this level is scarce, and only a few studies [30,47], including studies on humans [11,12], can be found. The underlying reasons for the absence of data could be related to certain difficulties while working with lung tissue, such as its spongy nature [47], heterogeneity in anatomical and functional structures (bronchia, bronchioles, alveoli), and cell type variety, with more than forty different existing cell types [28,37] that interact tightly to provide the right tissue architecture and functional capacity for effective gas exchange [28,37].

Current single-cell technologies allow for detangling tissue cell heterogeneity and analyzing its composition at the RNA level. However, because of the low quantity of cells obtained, establishing the lipid composition is highly challenging. MALDI-MSI offers a unique opportunity because, on the one hand, it is highly sensitive to lipid species, and, on the other hand, it provides a lateral resolution equivalent to the size of one to two cells. Following the single-cell approach, Wang et al. [48] isolated nine cell lineages from healthy lung tissue and established lineage-dependent differences in genes involved in fatty acid and glycerophospholipid metabolism. They showed that AA metabolism is downregulated, while alpha-linolenic acid (an essential fatty acid and precursor of DHA) is upregulated in the epithelial lineage compared to fibroblast and myeloid lineages [48], which is consistent with stromal AA enrichment and a lower DHA epithelial content (Figure 2c). Interestingly, AA- and DHA-containing phospholipids localize at the edges of airways in murine lungs, and although the specific tissue origin was not fully uncovered, the evidence pointed to the epithelial lining of airways [47].

Tissue malignization involves a profound metabolic reprogramming wherein lipid metabolism plays a prominent role, although the specific role of membrane phospholipid species is less established [49]. Tumor cells acquire the ability to thrive in a nutrient-poor environment by modifying their metabolic fluxes in response to a great demand for growth and proliferation. These changes extend to all cells in the tumor microenvironment that will need to compete with tumor cells for survival. Lipid metabolism exerts a pivotal role in this metabolic adaptation, and alterations of tumor lipid metabolism at the transcriptional level leading to the de novo lipogenesis activation were described [49,50]. Still, the specific role of membrane phospholipid species is less established.

Lung cancers usually originate from basal epithelial cells, located in the lower parts of the epithelium, where the broadest changes in lipids occurred, consistent with the central role of these cells [51] (Figure 3). PI and PE were the most affected classes, showing a robust increase in PI 38:4 and PE 38:4, which in the colon are related to the epithelial undifferentiation process occurring in adenomatous polyps [8] and chronic inflammation [10]. PC and PE AA species, particularly those of the immune cells, are considered the main source of lipid mediators, such as leukotrienes and prostaglandins, involved in the impairment of mucociliary clearance of particles or the promotion of bronchoconstriction and mucous secretion [47]. Furthermore, Marien et al. established lipid changes agreeing with ours in bulk lung cancer biopsies, revealing an increase in species with more than 36 carbons, which they linked to an altered expression in fatty acid elongases [11]. Consistently, Wang et al. used single-cell RNA-seq to demonstrate that pathways involved in the metabolism of glycerolipids, glycerophospholipids, fatty acid biosynthesis, and biosynthesis of unsaturated fatty acids were dysregulated in early-stage lung cancer in a cell-type-dependent manner [48]. Importantly for this study, Gouw et al. investigated gene expression changes in the lung of KRAS-activated mice, finding that, in addition to genes involved in de novo fatty acid synthesis, the activation also affects sphingolipid and PI metabolism genes [30]. Finally, we show that lung cancer affects not only the tumor lipid profile but also non-tumor tissue composition (Figure 5), which is consistent with the systemic impact cancer has on the homeostatic status.

Although 40–70% of patients with lung cancer are diagnosed with COPD [15,16], the exact link between them is uncertain, as both have similar causes, mainly smoking. COPD and lung cancer present oxidative damage, some genetic markers, and methylation patterns, as well as the inflammatory component of both diseases [51]. A better understanding of the relationship between these two diseases could benefit their simultaneous treatment. The most relevant change found herein was the increase in PE 38:4 in COPD and also smoker groups, which, as mentioned, is a precursor of lipid mediators such as LTB4 and PGE2, both raised in sputum of patients with COPD [52,53].

Finally, this study has two main limitations. First, the samples were analyzed exclusively in negative-ion mode; consequently, it was not feasible to obtain solid data for establishing a phosphatidylcholine (PC) profile, the most abundant membrane phospholipid. Fatty acid remodeling, the main cause of changes in lipid species content, is a phospholipid-dependent process [54]. In a previous study comparing non-tumor and malignant colon epithelia and stroma, the changes in PC profile followed similar trends to PI and PE, although less intense (except for PC 34:1) [8]. Nevertheless, epithelial and stromal PC profiles were specific to the tissue type, reinforcing the relevance of spatially resolved techniques. Conversely, we obtained unique information on phospholipids that are difficult to characterize by other methodologies that, despite being considered minor classes, are essential in cell signaling, such as phosphatidylinositol.

Secondly, there is the limitation in unequivocal lipid species identification, which is common to MSI lipidomic techniques and stems from the lack of a separation stage before the mass analysis. In the present study, all lipid species were carefully assigned using highly restrictive parameters. Furthermore, the resulting lipid profiles are comparable to those published and established with liquid chromatography-mass spectrometry (LC-MS) methodologies. Despite this, the presence of additional isobaric or isomeric lipid species cannot be ruled out, and consequently, the biological interpretation could be slightly affected by their presence. It is worth mentioning that, conscious of this constraint, the field is constantly improving the analytical processes so that this limitation is overcome [7]. In this context, the recent introduction of ion mobility cells into mass spectrometers helps the segregation of isobaric species [55]. Concurrently, Claes et al. introduced a new technique based on an ozone-induced dissociation to enable the high-resolution mapping of phospholipid isomers throughout relatively large areas [56]. Alternatively, it is possible to run MS/MS experiments directly from the section, although most current mass analyzers are still not capable of working in image mode and recording MS/MS experiments simultaneously. Finally, some MSI researchers use a hybrid approach, combining laser capture microdissection to select specific areas and analyze them with LC-MS (in this case, it is important to identify the impact of laser burning on phospholipids) [57].

## 5. Conclusions

Owing to improvements in MALDI-MSI instruments and protocols [54], not only is it becoming possible to reach subcellular resolutions but also to run kinetic approaches [55,56] or multiplexed immunohistochemistry [57]. Herein, we provide evidence sustaining the importance of spatially resolved molecular techniques to fully understand cell pathophysiology. Despite the intrinsic difficulties that lung tissue handling entails, the results strongly encourage tissue analysis at higher lateral resolutions to fully map the changes in lipid composition associated specifically with each lung cellular type and subtype. Undoubtedly, the rapid, constant advances in MSI techniques offer a unique opportunity to achieve this ambitious aim.

## Data Availability

The data that support the findings of this study are available from the corresponding author upon reasonable request.

## Supplementary Materials

The following supporting information can be downloaded at https://www.mdpi.com/article/10.3390/cancers17132160/s1: Figure S1. Classification and summary of the structural characteristics of membrane lipids in mammals; Table S1. Summary of all participants’ demographic and clinical data; Table S2. Comprehensive list of genes coding for proteins involved in phospholipid and sphingolipid metabolism found in lung cancer co-expressed modules; Table S3. Detailed lipid profile of the lipid segments. Reference [58] was cited in Supplementary Materials.

## Abbreviations

The following abbreviations are used in this manuscript:

AA	arachidonic acid
COPD	chronic obstructive pulmonary disease
DHA	docosahexaenoic acid
MALDI	matrix-assisted laser desorption/ionization
MSI	mass spectrometry imaging
PE	phosphatidylethanolamine
PE P	PE plasmalogens
PI	phosphatidylinositol
PS	phosphatidylserine
SM	sphingomyelin
