# Supplementary material for: Challenges and Advantages of Using Spatially Resolved Lipidomics to Assess the Pathological State of Human Lung Tissue
Source: Cancers (Basel). 2025 Jun 26;17(13):2160. doi: 10.3390/cancers17132160 (PMC12248445; doi:10.3390/cancers17132160)
Supplement: Supplementary file 1 [file cancers-17-02160-s001.zip › Supplementary Information Calvo I et al.pdf]

Challenges and Advantages of Using Spatially Resolved Lipidomics to Assess the Pathological State of Human Lung Tissue

Ibai Calvo [1], Albert Maimó-Barceló [2,3], Jone Garate [1], Joan Bestard-Escalas [2,3], Sergio Scrimini [2,3,4,5], Jaume Sauleda [2,3,4,5], Borja G. Cosío [2,3,4,5], José Andrés Fernández [1,*] and Gwendolyn Barceló-Coblijn [2,3,*]

[1] Department of Physical Chemistry, Faculty of Science and Technology, University of the Basque Country (UPV/EHU), 48940 Leioa, Spain; ibai.calvo@ehu.es (I.C.)

[2] Health Research Institute of the Balearic Islands (IdISBa), 07120 Palma, Spain; albert.maimo@idisba.es (A.M.-B.); juan.bestard@idisba.es (J.B.-E.); jaume.sauleda@ssib.es (J.S.); borja.cosio@ssib.es (B.G.C.)

[3] Research Unit, Hospital Universitari Son Espases, 07120 Palma, Spain

[4] Department of Respiratory Medicine, Hospital Universitari Son Espases, 07120 Palma, Spain

[5] Centro de Investigación Biomédica en Red in Respiratory Diseases (CIBERES), 28029 Madrid, Spain

* Correspondence: josea.fernandez@ehu.eus (J.A.F.); gwendolyn.barcelo@idisba.es (G.B.-C.)

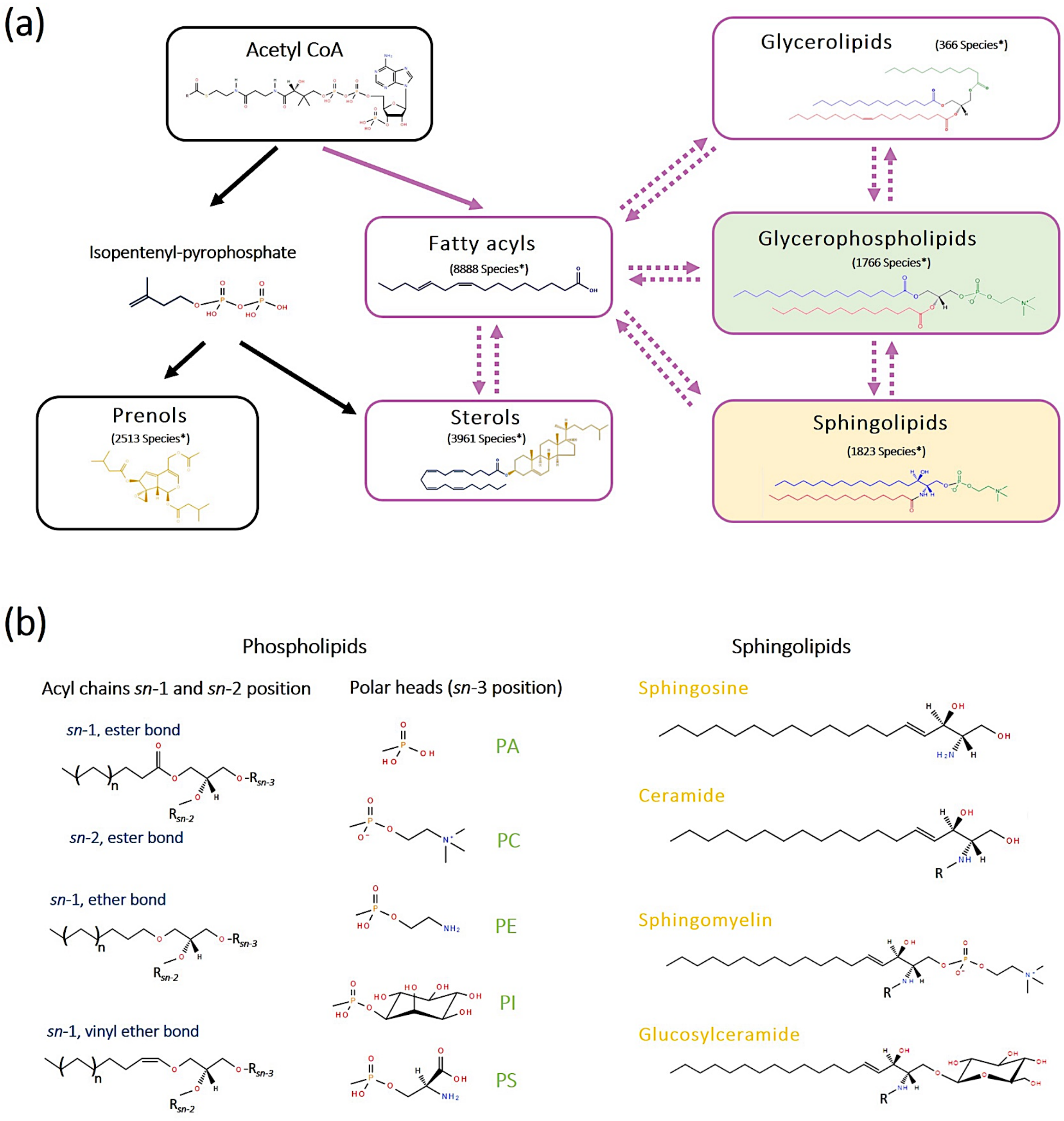

Figure S1. Classification and summary of the structural characteristics of membrane lipids in mammals. (a) **Lipid categories**. Metabolic interactions between the relevant lipid categories in mammals, including the number of lipid species defined so far in each class. Comprehensive classification including the following eight categories: fatty acids, glycerophospholipids (or phospholipids), sterols, sphingolipids, glycerolipids, and prenol lipids [1]. (Lipid Maps Structure Database®, last accessed Sept 2024). (b) **Structural elements of glycerophosphate- and sphingoid-based lipids. Phospholipids:**

On the left, the types of linkage established at sn-1 position between the fatty acid and the glycerophosphate backbone are shown. On the right, the most common polar heads (sn-3 position) found in mammalian cell membranes: phosphatidic acid (PA), phosphatidylcholine (PC), phosphatidylethanolamine (PE), phosphatidylserine (PS), and phosphatidylinositol (PI). **Sphingolipids:** The most frequent sphingoid base is sphingosine. Depending on the polar head linked -OH end of the ceramide, sphingomyelins (phosphocholine), cerebrosides (glucose, galactose…), and gangliosides (oligosaccharides or sialic acid) are generated. Adapted from Bestard-Escalas et al. 2019 [2].

1. Fahy E, Subramaniam S, Brown HA, Glass CK, Merrill AH, Murphy RC, et al. A comprehensive classification system for lipids. J Lipid Res. 2005;46:839–62.
2. Bestard-Escalas J, Maimó-Barceló A, Pérez-Romero K, Lopez DH, Barceló-Coblijn G. Ins and Outs of Interpreting Lipidomic Results. J Mol Biol. 2019;431:5039–62.

Table S1. Summary of all participants' demographic and clinical data.

Group	Patients (n=20)	Age (years)	Sex
Lung Cancer (Non-small cell lung cancer)	7	Mean 69.4<br>Median 71<br>Range 56 - 76	Male 6 (85.7%)<br>Female 1 (14.3%)
EPOC	4	Mean 72.2<br>Median 68<br>Range 67 - 85	Male 2 (50%)<br>Female 2 (50%)
Smokers	5	Mean 54.2<br>Median 57<br>Range 47 - 62	Male 0 (0%)<br>Female 5 (100%)
Non-smokers	4	Mean 53.7<br>Median 52.7<br>Range 45 - 65	Male 2 (50%)<br>Female 2 (50%)

Table S2. Comprehensive list of genes coding for proteins involved in phospholipid and sphingolipid metabolism found in lung cancer co-expressed modules.

Gene Symbol	Description	Modules
Phospholipid Metabolism HSA-1483257; PI Metabolism HSA-1483255		
ARF1	ADP-Ribosylation Factor 1	M1
CDIPT	CDP-Diacylglycerol--Inositol 3-Phosphatidyltransferase	M1
CDS2	CDP-Diacylglycerol Synthase 2	M1
CSNK2A1	Casein Kinase 2, Alpha 1 Polypeptide	M1
CSNK2A2	Casein Kinase 2, Alpha Prime Polypeptide	M1
CSNK2B	Casein Kinase 2, Beta Polypeptide	M1
DGAT1	Diacylglycerol O-Acyltransferase 1	M1
GDE1	Glycerophosphodiester Phosphodiesterase 1	M1
GPCPD1	Glycerophosphocholine Phosphodiesterase 1	M9
INPP5D	Inositol Polyphosphate-5-Phosphatase D	M1
LCLAT1	Lysocardiolipin Acyltransferase 1	M1
LPCAT2	Lysophosphatidylcholine Acyltransferase 2	M1
LPCAT3	Lysophosphatidylcholine Acyltransferase 3	M1
LPIN2	Lipin 2	M1
MBOAT2	Membrane Bound O-Acyltransferase Domain Containing 2	M1
PI4KA	Phosphatidylinositol 4-Kinase, Catalytic, Alpha	M1
PIK3C2A	Phosphatidylinositol-4-Phosphate 3-Kinase, Catalytic Subunit Type 2 Alpha	M1
PIK3C3	Phosphatidylinositol 3-Kinase, Catalytic Subunit Type 3	M1
PIK3R1	Phosphoinositide-3-Kinase, Regulatory Subunit 1 (Alpha)	M1
PIP4K2A	Phosphatidylinositol-5-Phosphate 4-Kinase, Type II, Alpha	M7
PIP5K1A	Phosphatidylinositol-4-Phosphate 5-Kinase, Type I, Alpha	M1
PITPNB	Phosphatidylinositol Transfer Protein, Beta	M1
PLEKHA1	Pleckstrin Homology Domain Containing, Family A (Phosphoinositide Binding Specific) Member 1	M1
PLEKHA2	Pleckstrin Homology Domain Containing, Family A (Phosphoinositide Binding Specific) Member 2	M1
PTDSS1	Phosphatidylserine Synthase 1	M1
RAB14	RAB14, Member RAS Oncogene Family	M1
RAB5A	RAB5A, Member RAS Oncogene Family	M1
STARD10	Star-Related Lipid Transfer Domain Containing 10	M1
STARD7	Star-Related Lipid Transfer Domain Containing 7	M1
Sphingolipid Metabolism Hsa-428157		
CERT1	Ceramide Transfer Protein 1	M1

ARSG	Arylsulfatase G	M1
ASAH1	N-Acylsphingosine Amidohydrolase (Acid Ceramidase) 1	M7
CERS6	Ceramide Synthase 6	M1
CTSA	Cathepsin A	M10
DEGS1	Delta(4)-Desaturase, Sphingolipid 1	M1
GBA	Glucosidase, Beta, Acid	M1
HEXB	Hexosaminidase B (Beta Polypeptide)	M1
ORMDL2	ORMDL Sphingolipid Biosynthesis Regulator 2	M1
ORMDL3	ORMDL Sphingolipid Biosynthesis Regulator 3	M1
PLPP2	Phospholipid Phosphatase 2	M12
PRKD1	Protein Kinase D1	M1
PRKD3	Protein Kinase D3	M1
PSAP	Prosaposin	M1
SGPP2	Sphingosine-1-Phosphate Phosphatase 2	M1
SPTSSA	Serine Palmitoyltransferase, Small Subunit A	M1
VAPA	VAMP Associated Protein A	M1
VAPB	VAMP (Vesicle-Associated Membrane Protein)-Associated Protein B And C	M1
